# News and Coming Events

**Published:** 1907-08-17

**Authors:** 


### NEWS AND COMING EVENTS.

A children's ward has been added to the Watton Cottage Hospital, erected in 1899, and was opened a few weeks ago by Mrs. Randolph, who has generously borne the total cost of its erection and furnishing. The new ward is an addition to the east wing of the building and has been built by Messrs. Waters and Son, of Watton, from plans prepared by Mr. Green, of Norwich.

The following nominations to lectureships during 1908 and 1909 have been announced by the Royal College of Physicians; Lumleian lecturer, Sir James Sawyer; Goulstonian lecturer, Dr. Herbert S. French; Fitzpatrick lecturer, Dr. Pye Smith; Oliver Sharpey lecturer, Professor Schaefer; Horace Dobell lecturer, Mr. L. S. Dudgeon; Croonian lecturer for 1909, Dr. Lazarus Barlow.

At the annual meeting of the North Devon Infirmary at Barnstaple, under the chairmanship of Lord Fortescue, an important alteration in the rules of the hospital was formally moved and sanctioned. The committee of management has for some considerable time had under discussion the question of excluding cases of enteric fever from the wards, and has finally moved an alteration of the rules to that effect.

Reports received before midnight on July 5, says the *Chicago Tribune,* showed that the number of persons dying as the direct result of the Fourth of July celebrations, was at least 45, and the number of injured exceeded 2,500. Since that time the figures have been greatly increased by reports of other casualties. In New York City wounds by pistols and firecrackers accounted for by far the larger number of fatalities.

Earl Cawdor, Treasurer of the London Homœopathic Hospital, Great Ormond Street, W.C., has received a cheque for £1,000 from Amy, Lady Tate, to endow the first bed for male patients in the new extension of the hospital, to be named the "Sir Henry Tate Bed." The hospital is appealing for £30,000 to extend the hospital on its own freehold site, and some £13,000 is still required to complete the fund.

Princess Louise Augusta of Schleswig-Holstein visited the Royal Free Hospital on August 3 and inspected the new mortuary chapel and the improvements to the mortuary, which have recently been carried out and the expense thereof borne by the Ladies' Association, of which her Highness is the President. At the present time two new operating theatres, with anæsthetising and sterilising rooms, are being erected; these works have been rendered necessary owing to the greatly increased surgical work of the hospital and in compliance with the urgent recommendations of the visitors of King Edward's Hospital Fund and the surgical staff of the hospital. The cost of these and other minor improvements will not be less than £10,000, and the committee hope that the benevolent public will assist them in raising this sum.

The eighth annual medical congress of Australasia will be held in Melbourne in October next. Professor H. Allen, lecturer in pathology at the University of Melbourne, will preside over the deliberations of the Congress.

A special committee of the Stafford Town Council has recommended the erection of an isolation hospital for the town. The estimated cost of the undertaking is £2,550. A site in Coton Field has been selected, and the plans and specifications will shortly be before the Council for final consideration.

At the half-yearly meeting of the Essex and Colchester Hospital, held last week, the Chairman, Colonel Tysson-Holroyd, drew attention to the scheme for the erection of a children's ward in connection with the institution. The plans provide for the acquisition of a completely furnished ward and accessories at an estimated cost of £2,000.

The Belfast Hospital for Diseases of the Skin has just acquired a new electrical and extern department, fully equipped with the most modern appliances for treatment. A mural tablet has been placed in the department, designating the additions as a memorial to the founder of the charity, Dr. Henry Purdom. The electrical apparatus was supplied by Messrs. Lizars.

The scheme for the erection of a children's hospital in Sunderland is progressing favourably. Already £15,000 of the £20,000 required for the building fund has been raised by local subscriptions, and an interesting feature of the appeal is the earnest way in which the working-classes have responded to it. An excellent site for the proposed institution has been presented to the Infirmary Committee by Mr. J. Pemberton.

A new course on School Hygiene, including lectures, demonstrations and practical work, has been arranged to begin on October 16 at University College. The course will be given by Professor Henry Kenwood and Dr. H. Meredith Richards. It is designed to meet the requirements of school teachers, school lecturers, and those qualifying for school inspectorships and for school medical officers. A certificate of proficiency will be granted to those who qualify themselves.

Her Grace the Duchess of Marlborough laid the foundation-stone of the new wing of the West Ham Hospital on July 27 last. The hospital itself was opened by the Duke of Westminster in 1890, and four years later the Passmore Edwards wing was added at the expense of Mr. Passmore Edwards. The new wing, of which the foundation-stone has just been laid, provides for a new casualty department, an operating theatre, two new wards (each containing 20 beds), and additional accommodation for the nursing staff. The estimate cost of the extensions is £20,000, towards which a sum of close upon £15,000 has already been raised.

